# Gut microbiota alters cardiac metabolism and immune system composition in viral myocarditis mice

**DOI:** 10.3389/fmicb.2026.1828423

**Published:** 2026-06-05

**Authors:** Lili Chen, Weifeng Wu, Qing Kong

**Affiliations:** Department of Cardiology, The First Affiliated Hospital of Guangxi Medical University, Nanning, China

**Keywords:** antibiotic, fecal microbiota transplantation, gut microbiota, immunity, metabolomics, viral myocarditis

## Abstract

**Background:**

The effects of the gut microbiota on the regulation of host physiology have recently garnered considerable attention, particularly in metabolism and the immune system. However, the role of the gut microbiota in viral myocarditis (VMC) remains to be fully elucidated.

**Methods:**

Balb/c mice were injected with CVB3 to induce VMC. VMC mice were treated with fecal microbiota transplantation (FMT) or antibiotics (ABX) to evaluate the therapeutic effects of these interventions. Echocardiography, HE, and Masson’s staining of the heart were used to assess cardiac function and pathological changes. 16S rDNA sequencing was conducted to explore alterations in gut microbial composition. UPLC-MS/MS-based metabolomics was used to detect disturbances of cardiac metabolic profiles. Flow cytometry was applied to analyze the dynamics of immune cell subsets, including M1, M2, Th1, Th2, Th17, and Treg cells. RT-PCR was performed to quantify cytokine expression levels in the heart.

**Results:**

FMT reduced cardiac inflammation and fibrosis, enhanced heart function, remodeled the structure of gut microbiota in VMC, and increased bacterial diversity, with an enrichment of p-Proteobacteria, the reduction of g-Pseudomonas, g-Streptococcus, and g-Ralstonia. Meanwhile, FMT induced alterations in cardiac metabolites in VMC, with enrichment of the steroid hormone biosynthesis pathway. A significant negative correlation was found between desoxycortone, corticosterone, 21-deoxycortisol, and cortodoxone with p_Spirochaetota and p_Kapabacteria. Furthermore, FMT reduced the proportions of M1 macrophages, Th1, and Th17 cells, as well as the cytokines TNF-a, IL-6, and IL-1β, and increased M2 macrophages and Treg cells. Regarding the role of antibiotics in VMC, our findings indicated that antibiotics altered the gut microbiota, myocardial metabolism, and immune response. Compared with antibiotics, FMT exerted a better effect on the alleviation of cardiac inflammation and fibrosis.

**Conclusion:**

In VMC mice, the gut microbiota, which mediates disturbances in cardiac metabolites and the host immune response, may contribute significantly to the development of cardiac inflammation and fibrosis. Furthermore, FMT may represent a promising therapeutic approach for VMC.

## 1 Introduction

Recently, a growing body of evidence has emerged regarding the important role of gut microbiota dysbiosis in the pathophysiology of cardiovascular diseases (Yu et al., 2025), especially in maintaining the host metabolism and the immune system homeostasis (Ishimwe et al., 2023), such as in ischemic heart disease (Fan et al., 2023), myocardial infarction (MI) (Tang et al., 2019), hypertension (O’Donnell et al., 2023). The immunomodulatory role of gut microbiota includes its impact on the composition, migration, and function of various immune cell subpopulations, and pathological abnormalities and inflammation may happen when this network is disturbed (Tang et al., 2019; Strati et al., 2021).

Fecal microbiota transplantation (FMT), which transfers fecal matter from a donor into the gastrointestinal tract of a recipient, aims to restore gut microbial imbalance toward eubiosis. For example, in recurrent *Clostridium difficile* infection, FMT is a safe treatment option and recommended by guidelines (Juul et al., 2018). Furthermore, FMT is considered a valuable tool to study the mechanistic link between dysbiosis and disease (Ishimwe et al., 2023). We recently found that there is a significant fluctuation of the gut microbiota in CVB3-induced mice with acute viral myocarditis (VMC)(Kong et al., 2023), but the role of the microbiota in AVMC was unknown, particularly for the host immunity and metabolism homeostasis. Therefore, in the current study, we used FMT to explore the association between gut microbiota and cardiac metabolism in VMC, and we detected macrophages (M1, M2), Th1, Th2, Th17, and Treg cells to explore the role of gut microbiota in the innate and adaptive immune system (Ramirez et al., 2020).

In addition to FMT, antibiotics (ABX) are considered a potential treatment for some diseases, through regulating intestinal microflora (Jia et al., 2019). For instance, in patients with Crohn’s disease, postoperatively, metronidazole reduced the endoscopic recurrence (Glick et al., 2019). Vancomycin disrupted the microbiota and led to prolonged loss of colonization resistance to *C. difficile* infection (Lewis et al., 2015). In myocardial infarction (MI), ABX altered the composition and metabolism of gut microbiota and worsened the outcome after MI (Tang et al., 2019). In inflammatory bowel disease (IBD), ABX causes intestinal macrophages to become hyperresponsive, produce excess inflammatory cytokines, and result in the dysregulation of Th1, Th2, and Th17 cells. This evidence demonstrated that ABX could affect the diversity and composition of gut microbiota and alter metabolic activity and immunity (Yuan et al., 2023). Whether ABX can be used to therapeutically manipulate the gut microbiota in VMC remains unknown.

Our previous study showed significant changes in T helper cells, such as Th17 cells; alterations of the gut microbiota; and metabolic profiles in CVB3-induced mouse VMC (Kong et al., 2023; Kong et al., 2022a; Kong et al., 2022b). We therefore hypothesized that the gut microbiota may have an important impact on the immune system and cardiac metabolome. To test this, we used FMT and ABX to examine the association among the gut microbiota, metabolome, and immune response in VMC mice.

## 2 Methods

### 2.1 Experimental animals

Our animal experiments were conducted in accordance with protocols approved by the Ethics Review Committee of the First Affiliated Hospital of Guangxi Medical University, China. BALB/c mice were purchased from Beijing Weitong Lihua Experimental Animal Technology (Beijing, China) [Permit Number: SCXK (JING) 2016–0006]. Four- to five-week-old male mice were used in the experiments and maintained under specific pathogen-free (SPF) barrier conditions at the Laboratory Animal Center of Guangxi Medical University. All mice were provided with standard drinking water and fodder and housed separately in individually ventilated cages according to their respective treatments. Coxsackievirus B3 (CVB3) was provided by the Microbiology Laboratory of the School of Basic Medical Sciences at Guangxi Medical University.

**Figure 1:**
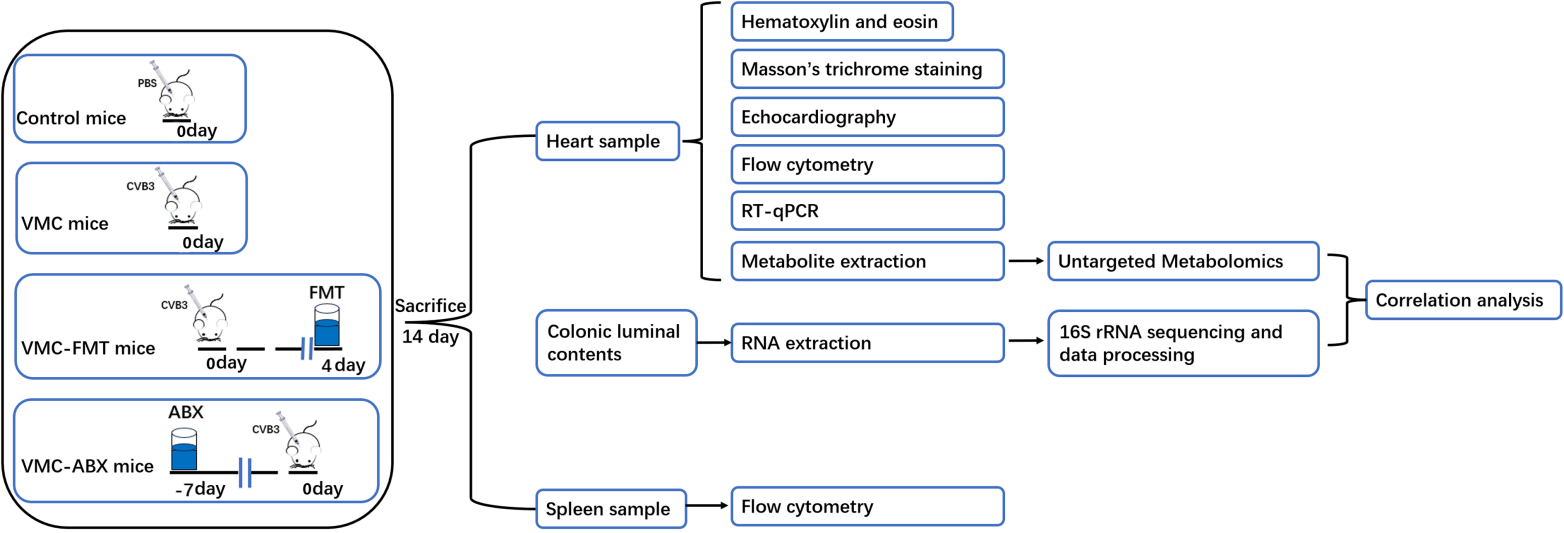
Schematic diagram of the experimental design.

A total of 26 mice were randomly divided into 4 groups (Figure 1): control group (*n* = 7), VMC group (*n* = 7), VMC-FMT group (*n* = 7), and VMC-ABX group (*n* = 5). Specifically, the control group ahd an intraperitoneal injection of 200 uL of PBS; the VMC group had an intraperitoneal injection of 200 uL of CVB3 virus dilution; and the VMC-FMT group: intraperitoneal injection of 200 uL of CVB3 virus dilution. On day 3, 200 uL of fecal supernatant from healthy mice was administered via anal perfusion once daily for 11 consecutive days. VMC-ABX group: intraperitoneal injection of 200 uL of CVB3 virus dilution. Prior to modeling, drinking water was supplemented with an antibiotic cocktail (0.25 mg/mL ampicillin, metronidazole, and neomycin, and 0.125 mg/mL of vancomycin) and sucralose (Splenda; 12 mg/mL) for 7 days. The day of intraperitoneal injection was defined as day 0. On day 14, echocardiography was performed to assess cardiac morphology and left ventricular ejection fraction (LVEF). All surviving mice were then sacrificed. Heart tissues were harvested for HE staining, Masson’s staining, PCR, flow cytometry of M1 and M2 macrophages, untargeted metabolomics, and histological analysis. Colonic contents were collected for DNA extraction and microbiome analysis. Spleens were isolated for flow cytometry to detect the proportion of Th1, Th2, Th17, and Treg cells.

### 2.2 Preparation of fecal bacterial solution from healthy mice

Cecal contents from healthy BALB/c mice were collected, then re-suspended in PBS, shaken on a plate shaker at 2500 rpm for 5 min, and centrifuged at 200 × *g* for 5 min. The supernatant was subsequently collected for FMT.

### 2.3 Histopathology

The ventricular tissues were collected sterilely and immersed in 4% paraformaldehyde overnight. After routine dehydration and embedding, myocardial tissue sections with a thickness of approximately 5 μm were prepared, followed by HE and Masson’s staining. Histopathological changes were observed under an upright optical microscope. The pathological score and myocardial fibrosis score were calculated by at least two investigators blinded to experimental grouping.

### 2.4 Echocardiography

On day 14 post-modeling, mice were anesthetized via intraperitoneal injection of 1.25% Avertin (approximately 0.2 mL/10 g). After hair removal and skin preparation, the chest was fully exposed. Transthoracic echocardiography (TTE) examination was performed using a MyLabTM Sat ultrasound imaging system. A 22 MHz probe was placed over the parasternal short-axis view, and M-mode images were acquired at the level of the left ventricular papillary muscles. Left ventricular end-systolic diameter (LVESD) and left ventricular end-diastolic internal diameter (LVEDD) and left ventricular end-diastolic diameter (LVEDD) were measured over three consecutive cardiac cycles. Left ventricular end-diastolic volume (LVEDV), end-systolic volume (LVESV), ejection fraction (LVEF), and fractional shortening (LVFS) were calculated using the Teichholz formula.

### 2.5 Flow cytometry

Hearts and spleens were aseptically harvested from mice, followed by digestion and erythrocyte lysis. Single-cell suspensions were obtained via centrifugation. For cardiac macrophages, cells were blocked and washed, then stained with anti-mouse FVS APC-Cy7, anti-mouse CD45 FITC, and anti-mouse F4/80 PerCP-Cyanine5.5. Intracellular staining was performed using a fixation/permeabilization kit with anti-mouse iNOS PE, anti-mouse arginase1 APC, and anti-mouse CD206 PE-Cy7. Cells were acquired using a BD FACSVerse flow cytometer. M1 macrophages were defined as CD45^+^F4/80^+^iNOS^+^, and M2 macrophages as CD45^+^F4/80^+^Arg1^+^ or CD45^+^F4/80^+^CD206^+^. For intracellular cytokine staining (ICS) of Th cells, splenocytes were stimulated with PMA (100 ng/mL), ionomycin (1.5 μg/mL), and brefeldin A (BFA, 15 μg/mL) for 4–6 h at 37 °C with 5% CO₂. Cells were then harvested, blocked, and stained with surface markers (FVS APC-Cy7, CD4 FITC), followed by permeabilization and staining with anti-mouse IL-17A PerCP-Cy5.5, anti-mouse IL-4 PE, and anti-mouse IFN-*γ* APC. Th1, Th2, and Th17 cells were defined as CD4^+^IFN-γ^+^, CD4^+^IL-4^+^, and CD4^+^IL-17A^+^, respectively. For splenic Treg cells, single-cell suspensions were blocked and stained with anti-mouse CD4 PerCP-Cy5.5 and anti-mouse CD25 PE. Cells were fixed and permeabilized for intracellular staining with anti-mouse FoxP3 APC. Treg cells were identified as CD4^+^CD25^+^FoxP3^+^. Data were analyzed using FlowJo v10 software.

### 2.6 RT-qPCR

Sterilely collect mouse myocardial tissue, add TRIzol, perform magnetic bead grinding in a tissue homogenizer, and lyse the cells. Then, add chloroform and isopropanol, and use RNase-free reagents in accordance with the instructions to extract total RNA. The PrimeScript™ RT reagent Kit with gDNA Eraser was used to remove genomic DNA and reverse-transcribe RNA into cDNA. mRNA primers were designed and synthesized by Shenggong Biotechnology Co., Ltd. The primer sequences are listed as follows: GAPDH: TGTGTCCGTCGTGGATCTGA/TTGCTGTTGAAGTCGCAGGAG; IL-1β: TCGCAGCAGCACATCAACAAGAG/AGGTCCACGGGAAAGACACAGG; IL-6: CTCCCAACAGACCTGTCTATAC/CCATTGCACAACTCTTTTCTCA; TNF-*α*: ATGTCTCAGCCTCTTCTCATTC/GCTTGTCACTCGAATTTTGAGA; IL-10: CCGAGATGCCTTCAGCAGAGT/ GGAGTTCACATGCGCCTTGAT; Arg1: CGGCTTGCGAGATGTGG/TAGCCGGGGTGAATACTGG; CD206: GTGAACGGAATGATTGTGTAG/TTGGTTGTAATGGATGAGTGT; CD86: GGGGGATCCATGGGCTTGGCAATCCTTAT/TCGGGTGACCTTGCTTAGACGTGCAGG. Subsequently, quantitative real-time PCR was performed using TB Green Premix Ex Taq II (Tli RNaseH Plus), and gene expression was detected with the ABI 7500 Real-Time PCR System.

### 2.7 16S rRNA gene sequencing and data analysis

DNA extraction from colonic luminal contents, 16S rRNA gene amplification, purification, library preparation, and sequencing data processing were performed on the NovaSeq 6,000 platform. Briefly, paired-end reads were demultiplexed based on unique barcodes and trimmed to remove barcode and primer sequences. The V3–V4 region of the 16S rRNA gene was amplified using primers 341F (5′-CCTACGGGNGGCWGCAG-3′) and 806R (5′-GGACTACHVGGGTWTCTAAT-3′). PCR conditions included initial denaturation at 98 °C for 1 min, followed by 30 cycles of 98 °C for 10 s, 50 °C for 30 s, and 72 °C for 30 s, with a final extension at 72 °C for 5 min. Quality control involved filtering low-quality reads (Q ≥ 20) and removing chimeric sequences using UCHIME. Paired-end reads were merged using FLASH software (V1.2.7^1^) (Magoč and Salzberg, 2011) to obtain raw tags. After quality filtering and quality control (Bokulich et al., 2013; Caporaso et al., 2010), clean tags were compared with the SILVA database^2^ using the UCHIME algorithm (UCHIME Algorithm^3^) (Edgar, 2013) to detect and remove chimera (Haas et al., 2011), yielding effective tags. Sequence analysis was performed using Uparse software (Uparse v7.0.1001^4^) (Li et al., 2013). Sequences with ≥97% similarity were clustered into operational taxonomic units (OTUs). Taxonomic annotation of representative sequences was performed using the SILVA database^5^ (Quast et al., 2012) based on the Mothur algorithm at the kingdom, phylum, class, order, family, genus, and species levels (threshold set at 0.8 ~ 1). Multiple sequence alignment was performed using MUSCLE (Version 3.8.31^6^) to analyze phylogenetic relationships (Edgar, 2004). Alpha diversity (Shannon and Simpson indices) was calculated using QIIME (Version 1.9.1) to assess species richness and evenness. Beta diversity was evaluated using weighted and unweighted UniFrac distances, calculated by QIIME software (Version 1.9.1). Principal Coordinate Analysis (PCoA) was visualized using the WGCNA stats and ggplot2 packages in R software (Version 4.5.0). Significant differences in taxonomic abundance were analyzed using R. Linear discriminant analysis effect size (LEfSe) was performed to identify biomarkers characterizing differences between groups (Segata et al., 2011).

### 2.8 Metabolomic analysis

As described in our previous study (Kong et al., 2023), 20 mg of heart sample was taken for heart preparation. Then the supernatant was analyzed using a high-performance liquid chromatography-electrospray tandem mass spectrometry (LC-ESI-MS/MS) system (UPLC, ExionLC AD; MS, QTRAP® 6,500 + System, Sciex) to obtain metabolites. Metabolomics analysis was performed using UPLC-MS/MS with an ESI source operating in both positive and negative ion modes. Mass calibration was carried out using 10 and 100 μmol/L polypropylene glycol solutions to ensure mass accuracy. The ion spray voltage was set to 5,500 V (positive) and −4,500 V (negative), with a source temperature of 500 °C. Using the self-built target standard database MWDB (Metware database), which is widely used in the targeted UPLCMS/MS platform of Metware Biotechnology Co., Ltd. (Wuhan, China), metabolites were annotated. Unsupervised PCA (principal component analysis) was performed using the statistics function prcomp within R (www.rproject.org). The R package MetaboAnalystR was used to generate the VIP value. According to VIP > =1 and *p* < 0.05, significantly changed metabolites among the four groups were identified. These differential metabolites were then annotated in the KEGG database,^7^ classified according to KEGG pathway types,^8^ and a hypergeometric test was used to identify significantly enriched pathways.

### 2.9 Analysis of data

All data were analyzed using SPSS 26.0 software. Normally distributed data with homogeneous variance were expressed as mean ± standard deviation. Student’s t-test was used for two-group comparisons, and one-way ANOVA followed by an LSD t-test was applied for multiple comparisons. Welch’s corrected ANOVA and Games-Howell *post hoc* test were adopted for data with unequal variance. Non-normally distributed data were presented as a median [P25, P75]. The Kruskal–Wallis H test was used for overall comparison, and Dunn’s test with Bonferroni correction was performed for pairwise comparisons. Spearman’s rank correlation analysis was conducted to evaluate the correlation between cardiac metabolites and gut microbiota. A correlation with |r| ≥ 0.8 and *p* < 0.05 was regarded as statistically significant. A *p*-value of < 0.05 was considered statistically significant in all tests.

## 3 Result

### 3.1 FMT ameliorated the pathology of the heart

**Figure 2:**
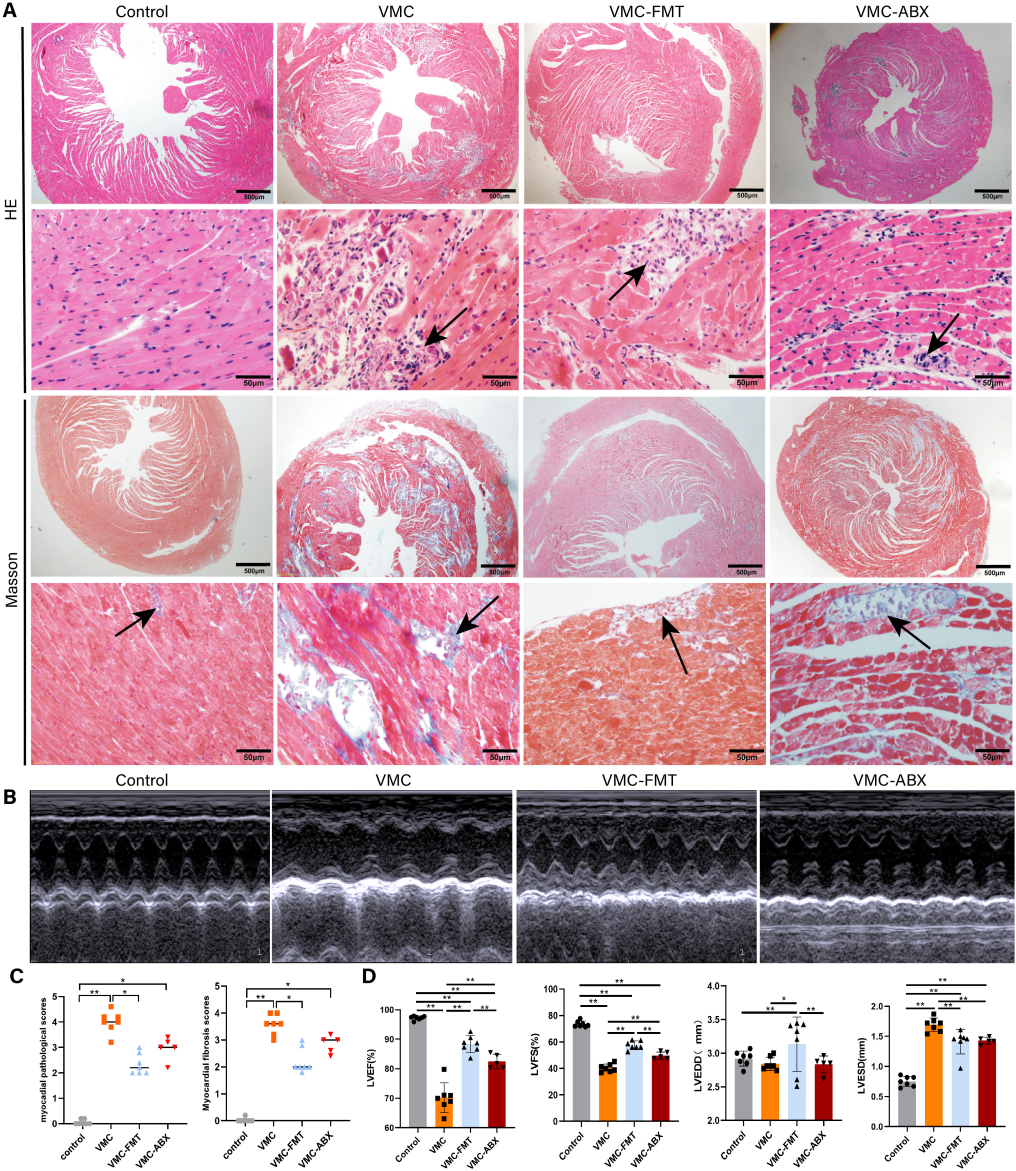
Myocardial histopathological changes and echocardiography in different groups. **(A)** Representative H&E and Masson’s trichrome staining images of myocardial tissues from the control, VMC, VMC-FMT, and VMC-ABX groups (*n* = 5–7/group), original magnification: 4 × and 40×; scale bars = 500 μm and 50 μm. **(B)** Representative echocardiographic images of the four groups. **(C)** Bar graphs show the statistical results of the pathological scores and cardiac collagen volume fractions. **(D)** Statistical analysis of LVEDV, LVESV, LVEF, and LVFS (*n* = 5–7/group). Data are presented as the means ± SEM. FMT, fecal microbiota transplantation; ABX, antibiotics; CVB3, coxsackievirus B3; H&E, hematoxylin and eosin; LVEDV, left ventricular end-diastolic volume; LVESV, left ventricular end-systolic volume; LVEF, left ventricular ejection fraction; LVFS, left ventricular fractional shortening; SEM, standard error of mean. *p < 0.05, **p < 0.01.

The evaluation of myocardial inflammation and fibrosis is shown in Figures 2A,C. In the control group, no inflammatory infiltration, cardiomyocyte necrosis, or collagen deposition was observed. Compared with the control group, the VMC group exhibited extensive inflammatory cell infiltration, cardiomyocyte necrosis, and collagen deposition. The pathological score (4 [3.6004, 4.3996]) and fibrosis score (3.6 [3.2266, 3.9163]) were markedly increased (*p* < 0.01), indicating successful establishment of the VMC model. Compared with the VMC group, the VMC-FMT group presented significantly reduced inflammatory infiltration, cardiomyocyte necrosis, and collagen accumulation. Consistently, the pathological score (2.2 [1.9621, 2.7236]) and fibrosis score (2.4 [2.0623, 2.7377]) in the VMC-FMT group were significantly lower than those in the VMC group (all *p* < 0.05). Regarding the effect of antibiotics (ABX) in VMC, the pathological score (3 [2.3937, 3.5263]) and fibrosis score (3 [2.4319, 3.2481]) in the VMC-ABX group were lower than those in the VMC group, but the differences were not statistically significant (all *p* < 0.05). Collectively, FMT intervention effectively alleviated myocardial inflammation and fibrosis in VMC mice, thereby exerting cardioprotective effects.

To compare the left ventricular size and function among different groups, we performed TTE on day 14 (Figure 2B). As shown in Figure 2D, compared with the control group, LVESD in the VMC group and VMC-FMT group was significantly increased (*p* < 0.05 for both). Compared with the VMC group, the LVESD in the VMC-FMT group was significantly reduced (*p* < 0.05); LVEDD in the VMC-FMT group was significantly larger than that in the VMC group and the control group (*p* < 0.05 for both); LVEF and LVFS in the VMC group and the VMC-FMT group were significantly lower than those in the control group (*p* < 0.05 for both), while LVEF and LVFS in the VMC-FMT group were significantly higher than those in the VMC group (*p* < 0.05 for both). Compared with the VMC mice, the VMC-ABX group exhibited higher LVESD, LVFS, and LVEF (all *p* < 0.01). LVEF in the VMC-ABX group was lower than that in the VMC-FMT group (*p* < 0.01). These results revealed that FMT is superior to ABX in improving cardiac function.

### 3.2 FMT and ABX remodel the structure of gut microbiota in VMC

**Figure 3:**
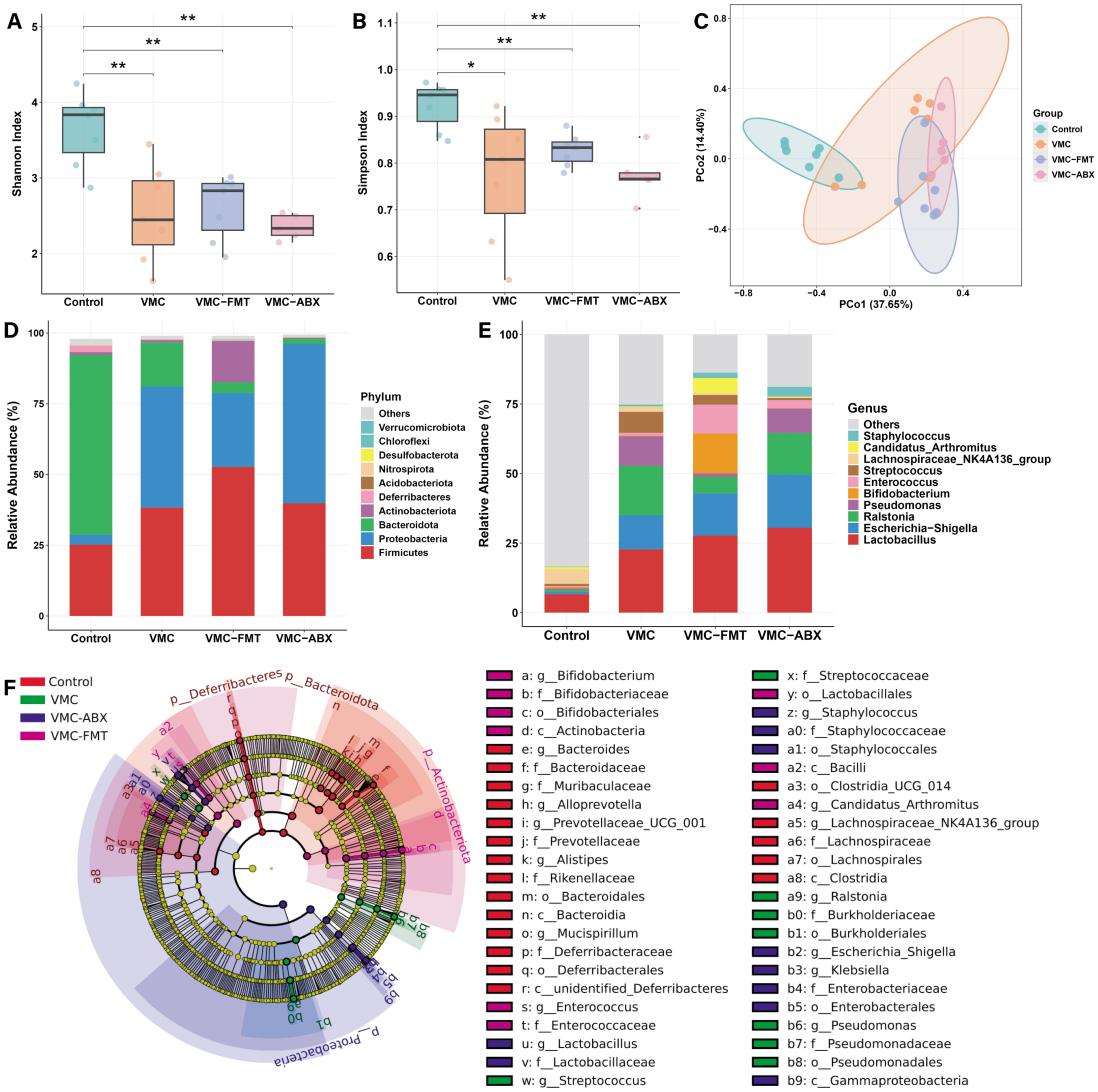
Analysis of gut microbiota structure among different groups. **(A,B)** Alpha diversity indices, including Shannon and Simpson; **(C)** Principal coordinate analysis (PCoA) of beta diversity based on Bray–Curtis dissimilarity. **(D,E)** Mean relative abundance of gut microbiota at the phylum and genus levels. All genera with relative abundance < 0.1% are reported together and labeled as “Others.” **(F)** Cladogram indicates the phylogenetic distribution of microbiota correlated with the four groups. *p < 0.05, **p < 0.01.

**Figure 4:**
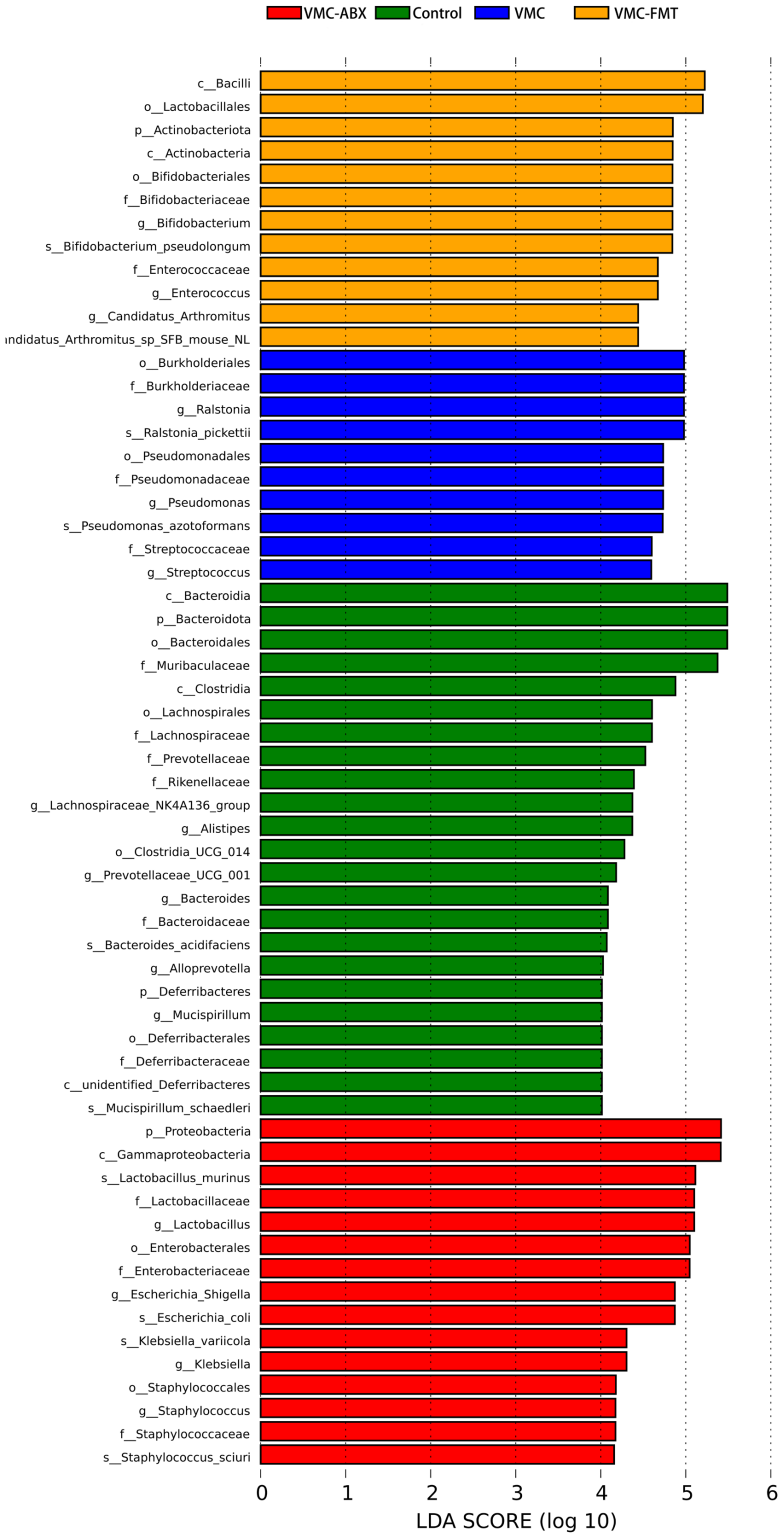
Linear discriminant analysis (LDA) of four groups. LDA identified gut bacterial taxa with significantly different abundances among the groups.

We characterized differences in gut microbiota structure using 16S rDNA sequencing. First, species-accumulation boxplots confirmed sufficient sampling depth (Supplementary data). Shannon and Simpson diversity indices in the control group were higher than those in the other three groups (Figures 3A,B) (all *p* < 0.05). Second, principal coordinate analysis (PCA) revealed distinct clustering of the gut microbiota in the control group compared to the other groups (Figure 3C), although the VMC group showed a high degree of overlap with the VMC-FMT and VMC-ABX groups. Third, we compared taxonomic composition across groups (Figures 3D,E). At the phylum level, the relative abundance of *Bacteroidota* was significantly lower in the other three groups compared to the control group. Compared with the VMC group, the VMC-FMT group exhibited a lower abundance of Proteobacteria and a higher abundance of Firmicutes. Notably, the VMC-FMT group had the highest abundance of p_Actinobacteriota, while the VMC-ABX group had the highest abundance of Proteobacteria. At the genus level, the VMC-FMT group had a composition that was more similar to the control group, i.e., with a higher proportion of Pseudomonas, Streptococcus, and Ralstonia in the VMC group, but those changes were reversed by the VMC-FMT group. Moreover, the abundance of g-Escherichia-Shigella was higher than the other three groups. Evolutionary branching plots demonstrated that specific bacterial taxa in the VMC-FMT group clustered closely with those in the control group (Figure 3F). Furthermore, LEfSe analysis identified specific biomarker taxa for each group (Figure 4). The top four-ranked colonies in the control group were c-Bacteroidia, p-Bacteroidota, o-Bacteroidales, and o-Muribaculaceae. The top four taxa in the VMC group were o-Burkholderiales, f-Burkholderiaceae, g-Ralstonia, and s-Ralstonia-pickettii. The top four taxa in the VMC-FMT group were c-Bacilli, o-Lactobacillales, p-Actinobacteriota, and c-Actinobacteria. The top four-ranked colonies in the VMC-ABX group were p-Proteobacteria, c-Gammaproteobacteria, s-Lactobacillus-murinus, and f-Lactobacillaceae. In conclusion, FMT and ABX significantly altered the gut microbiota composition in VMC mice. Notably, FMT remodeling partially restored the microbiota structure toward that of the healthy control group.

### 3.3 Gut microbiota influences myocardial inflammation through changes in cardiac metabolites

**Figure 5:**
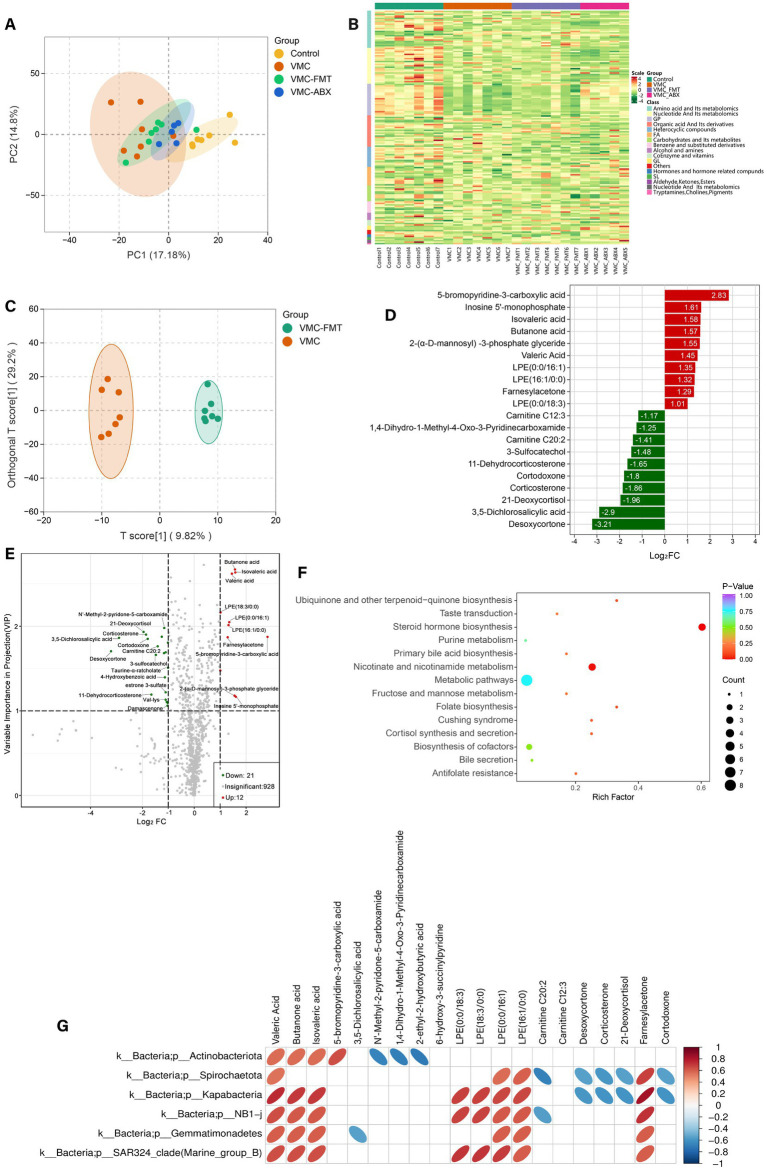
Cardiac metabolism analysis in VMC and VMC-FMT groups. **(A)** Principal component analysis (PCA) of myocardial metabolites across the control, VMC, VMC-FMT, or VMC-ABX groups. **(B)** Heatmap shows the relative abundance of myocardial metabolites in the four groups. **(C–F)** Comparison of metabolomics data between the VMC and VMC-FMT groups: OPLS-DA analysis **(C)**, top FC scores of the important discriminatory metabolites obtained from the OPLS-DA models **(D)**, volcano plot of the differentially accumulated and significantly changed metabolites **(E)**, and differential metabolites enriched in 20 KEGG pathways **(F)**. **(G)** Heatmap of Spearman’s correlations between the metabolomics of the VIP top 20 and gut microbiota with statistical differences at phylum taxa. The red ellipse indicates a positive correlation, while the blue indicates a negative correlation. The higher the absolute correlation value, the thinner the ellipse. No color indicates that the *p* > 0.05. Heat maps are drawn using the R software and the corrplot package.

**Figure 6:**
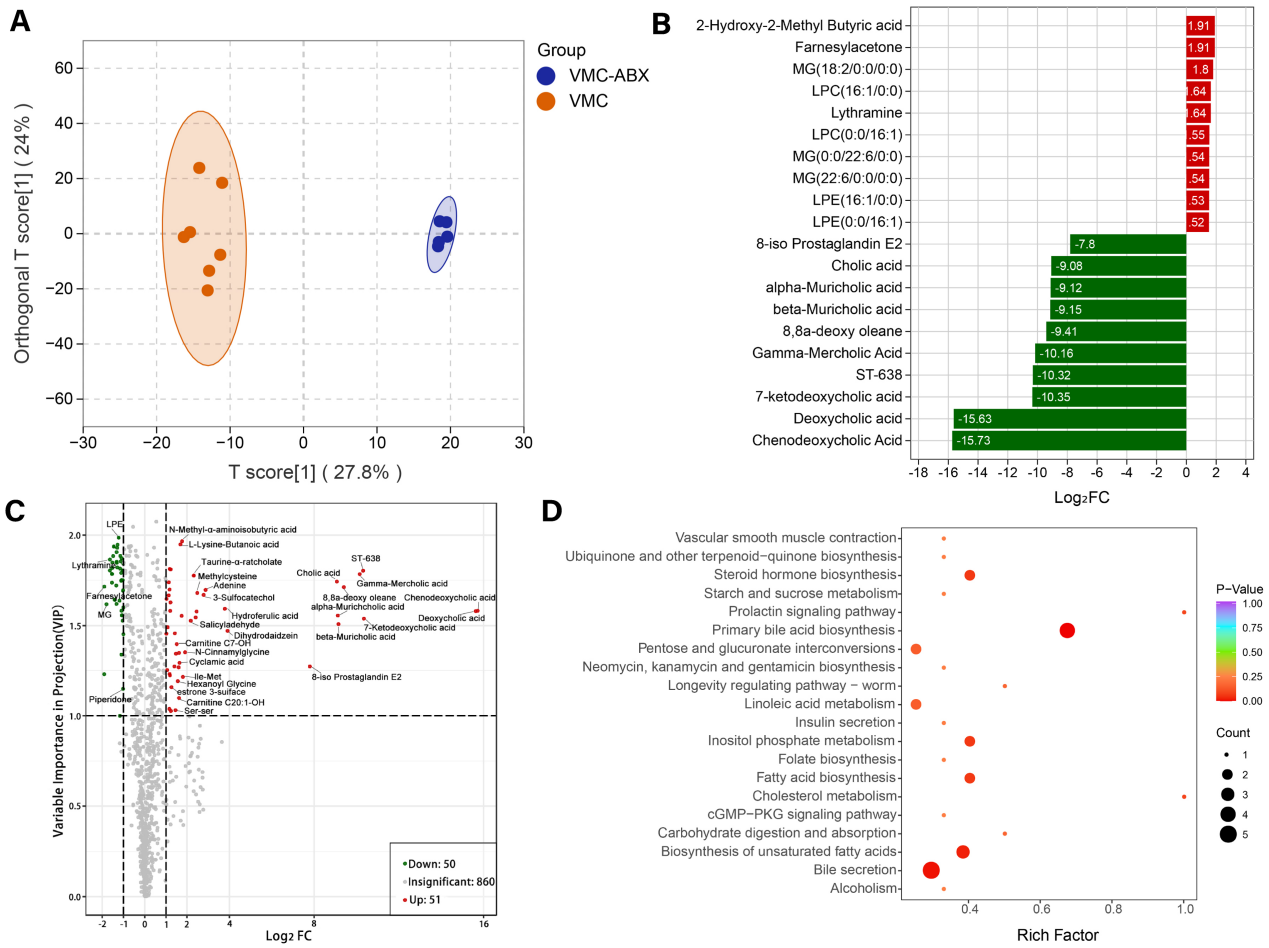
Cardiac metabolism analysis of the VMC and VMC-ABX groups **(A–D)**. Comparison of metabolomics data between the VMC and VMC-ABX groups: OPLS analysis **(A)**; top FC scores of the important discriminatory metabolites **(B)**; volcano plot of the differentially accumulated and significantly changed metabolites **(C)**; and differential metabolites enriched in 20 KEGG pathways **(D)**.

Principal component analysis showed large differences between the control group and the other three groups (Figure 5A). While the distribution of cardiac metabolites in the VMC group overlapped to a high degree with that in the VMC-FMT and VMC-ABX groups, this trend was similar to that of the gut microbiota. Heatmap of metabolomics data was consistent with the principal component analysis (Figure 5B). We further compared the differences between the VMC and VMC-FMT groups to clarify the effect of VMC-FMT (Figures 5C–F). OPLS-DA analysis revealed significant differences between them (Figure 5C). The top fold change (TopFc) metabolites between the VMC and VMC-FMT groups were desoxycortone, 3,5-dichlorosalicylic acid, 21-deoxycortisol (Figure 5D). The volcano plot showed that 21 metabolites were upregulated and 12 metabolites were downregulated in the VMT-FMT group compared to the VMC group (Figure 5E), which were mainly enriched in the steroid hormone biosynthesis pathways (Figure 5F). We then correlated the top 20 metabolites with VIP values and gut microbiota that had statistical differences at the phylum level and observed a significant positive correlation between desoxycortone, corticosterone, 21-deoxycortisol, and cortodoxone with p_Spirochaetota and p_Kapabacteria (Figure 5G). Then, to explore the role of antibiotics in VMC, we compared cardiac metabolites between the VMC and VMC-ABX groups (Figures 6A–D). OPLS-DA analysis found significant differences between them (Figure 6A). TopFc metabolites between the VMC and VMC-ABX groups were chenodeoxycholic acid and deoxycholic acid (Figure 6B). The volcano plot showed that 51 metabolites were upregulated and 50 metabolites were downregulated in the VMC-ABX group compared to the VMC group (Figure 6C), which were mainly enriched in the primary bile acid biosynthesis pathways (Figure 6D). In summary, both FMT and ABX exerted a significant effect on cardiac metabolism.

### 3.4 FMT and ABX-regulated macrophage polarization

**Figure 7:**
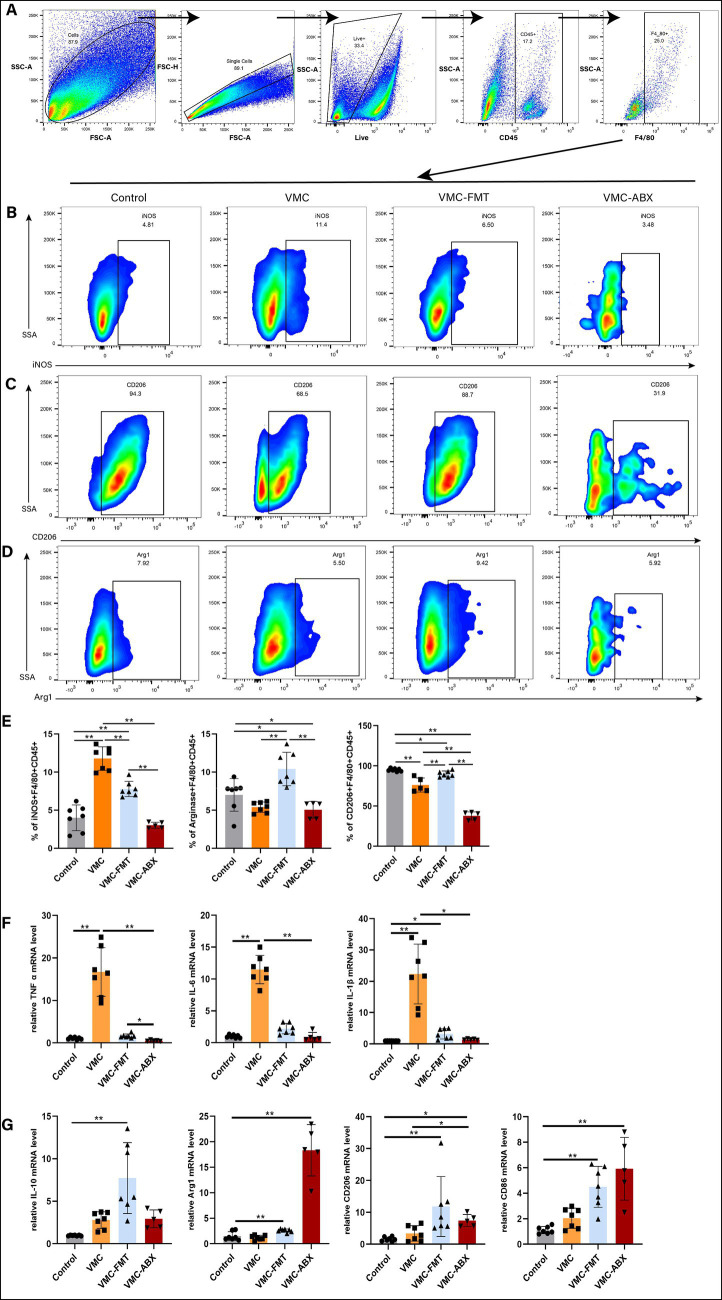
Levels of M1, M2 cells, and their associated cytokines in different groups. **(A)** Representative gating strategy for macrophages by flow cytometry. **(B)** Representative flow cytometry images of the frequencies of cardiac M1 cells in four groups (control, VMC, VMC-FMT, and VMC-ABX, *n* = 5–7/group). **(C,D)** Frequencies of cardiac M2 cells in different groups. (D, F) Statistical results of the frequencies of M2. **(E)** The bar graph indicates the statistical results of the frequencies of M1 and M2. **(F,G)** Levels of cytokines that are associated with M1 and M2: TNF-*α*, IL-6, IL-1β, IL-10, Arg1, CD206, and CD86. *p < 0.05 and **p < 0.01.

On day 14, cardiac tissues were harvested and processed into single-cell suspensions for flow cytometric analysis. The proportion of M1 macrophages (CD45^+^F4/80^+^iNOS^+^) was quantified (Figures 7A,B,E). Compared with the control group, the number of M1 cells in the VMC and VMC-FMT groups was significantly increased (*p* < 0.01). However, compared with the VMC group, the proportions of M1 cells in the VMC-FMT group significantly decreased (*p* < 0.01). Regarding M2 macrophages (M2: CD45^+^F4/80^+^Arg1^+^ or CD45^+^F4/80^+^iNOS^+^, CD45^+^F4/80^+^CD206^+^) (Figures 7A,C–E), the proportions of CD45^+^F4/80^+^Arg1^+^ cells were significantly increased in the VMC-FMT group compared to the control group (*p* < 0.05), whereas they were decreased in the VMC group (*p* > 0.05). Compared with the VMC group, the VMC-FMT group exhibited a significant increase in CD45^+^F4/80^+^Arg1^+^ cells (*p* < 0.01). Compared with the control group, the proportions of CD45^+^F4/80^+^CD206^+^ + cells were decreased in the VMC and VMC-FMT (p < 0.05), and this increase was more pronounced in the VMC-FMT group compared to the VMC group (*p* < 0.01) (Figures 7D,E). Additionally, compared with the VMC group, we detected lower proportions of M1 cells and M2 cells (*p* < 0.01) in the VMC-ABX group (Figures 7B,E). Overall, both FTM and ABX reduced the proportions of M1 cells. M2 cell levels were increased by FTM but decreased by ABX.

### 3.5 Changes of cytokines in the FMT groups and the ABX groups

As shown in Figures 7F,G, compared with the control group, the mRNA expression levels of pro-inflammatory factors TNF-*α*, IL-6, and IL-1β in the myocardial tissues of the VMC group were significantly increased (all *p* < 0.01). Compared with the VMC group, the levels of TNF-α, IL-6, and IL-1β of the VMC-FMT group were lower, and the level of anti-inflammatory factor IL-10 was higher, but the differences were not statistically significant. Further analysis of the polarization markers of macrophages revealed that, compared with the VMC group, the levels of Arg1, CD206, and CD86 in the VMC-FMT group mice were increased, while the differences were not statistically significant. Additionally, compared with the VMC group, the levels of TNF-α, IL-6, IL-1β, and CD206 in the VMC-ABX group were higher (all *p* < 0.01). In summary, both FMT and ABX reduced the levels of the pro-inflammatory factors TNF-α, IL-6, and IL-1β. Meanwhile, FMT markedly elevated the expression of the anti-inflammatory factor IL-10.

### 3.6 FMT and ABX affect the levels of Th1, Th2, Th17, and Treg cells

**Figure 8:**
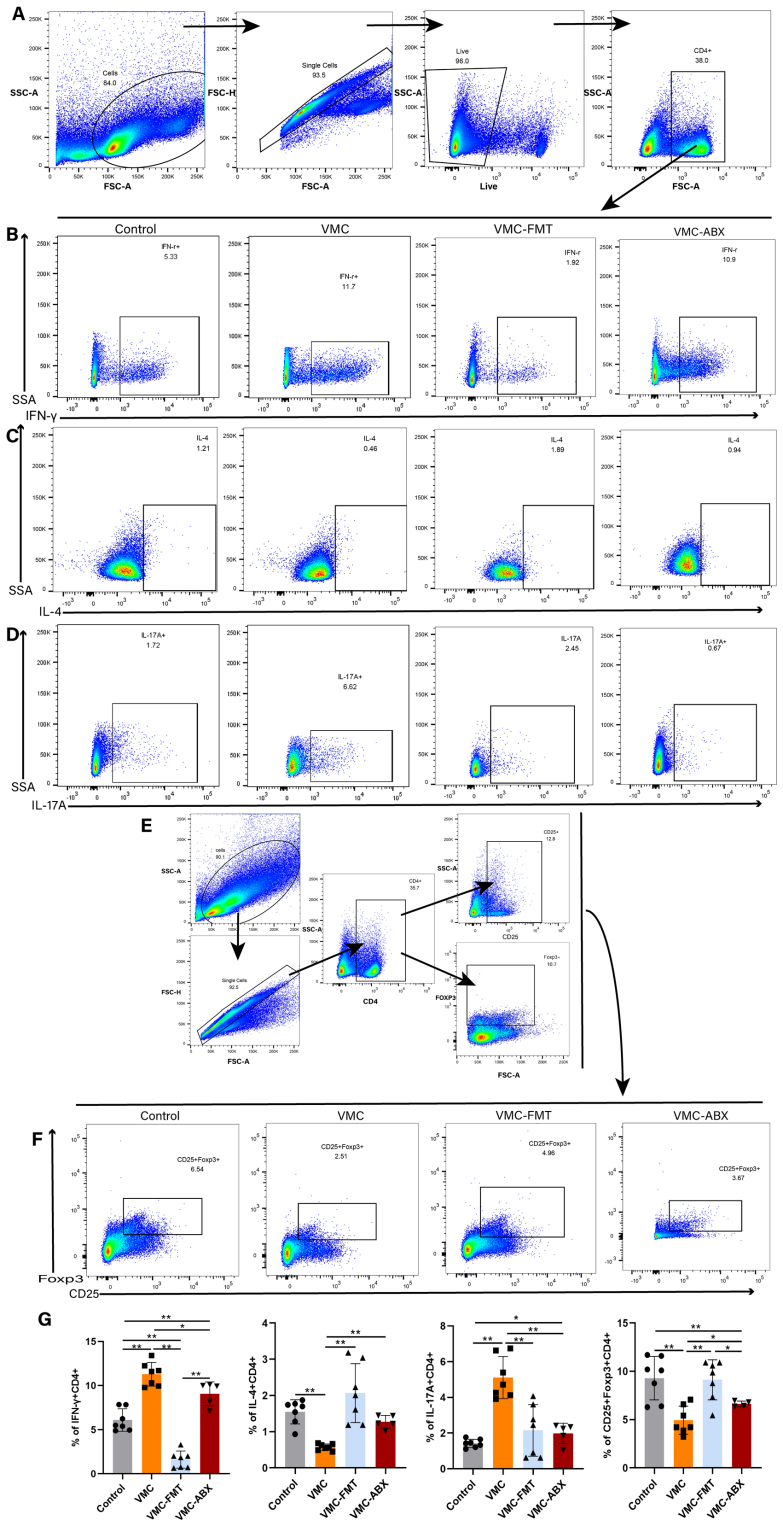
Frequencies of Th1, Th2, Th17, and Treg cells in four groups. **(A)** Representative gating strategy for Th1, Th2, and Th17 by flow cytometry. **(B)** Representative flow cytometry images of the frequencies of Th1 cells in different groups. **(C)** Representative flow cytometry images of the frequencies of Th2 cells in different groups. **(D)** Representative flow cytometry images of the frequencies of Th17 cells in different groups. **(E)** Representative gating strategy for Treg by flow cytometry. **(F)** Representative flow cytometry images of the frequencies of Treg cells in different groups. **(G)** The bar graph shows the statistical frequencies of Th1, Th2, Th17, and Treg cells. *p < 0.05, **p < 0.01.

As shown in Figures 8A,B,G, compared with the control group, the level of Th1 cells (CD4^+^CD25^+^IFN-γ^+^) in the VMC group was significantly increased, and the level of Th1 cells in the VMC-FMT group was lower than that in the VMC group (*p* < 0.01). Additionally, as shown in Figures 8A,C,G, compared with the control group, the level of Th2 cells (CD4^+^IL-4^+^) in the VMC group was significantly decreased (*p* < 0.01), and the level of Th2 cells in the VMC-FMT group was significantly higher than that in the VMC group (*p* < 0.01). Furthermore, we examined Th17 cells (CD4^+^IL-17A^+^) and Treg cells (CD4^+^CD25^+^FoxP3^+^). As shown in Figures 8A,D,G, compared with the control group, the level of Th17 cells in the VMC group was significantly increased (*p* < 0.01), while the level of Th17 cells in the VMC-FMT group was significantly lower than in the VMC group (*p* < 0.01). Regarding Tregs (Figures 8E–G), the level in the VMC-FMT group was significantly increased compared to the VMC group (*p* < 0.01). In the VMC-ABX group, we detected significantly lower proportions of Th1 and Th17 cells, alongside higher levels of Th2 and Treg cells, compared to the VMC group. In conclusion, FMT significantly reduced the proportions of Th1 and Th17 cells while increasing the levels of Th2 and Treg cells. ABX markedly elevated the proportion of Th1 cells.

## 4 Discussion

In our preliminary study, evidence of microbial dysbiosis and metabolite dysregulation was found in CVB3-induced VMC. These data suggested that gut microbiome dysbiosis and metabolic disturbance were involved in the pathogenesis of VMC. However, the precise association between VMC and gut microbiota has yet to be fully elucidated. In the current study, we treated mice with fecal microbiota transplantation (FMT) and antibiotics (ABX) to assess the effects of gut microbiota on VMC. Our results indicated that both FMT and ABX ameliorated cardiac inflammation and fibrosis and improved cardiac function, with FMT superior to ABX. In conclusion, FMT is a promising therapeutic approach for viral myocarditis.

FMT has been developed to treat various diseases such as obesity, metabolic syndrome, and diabetes (Ng et al., 2022; Mocanu et al., 2021; Surawicz et al., 2013). Lots of current research attempts to investigate the effectiveness and safety of FMT in cardiovascular disease, including pulmonary arterial hypertension (Chen et al., 2022), heart failure, and ventricular remodeling (Shi et al., 2023), although these studies rely predominantly on animal models (Yu et al., 2025). Beyond ethical concerns, FMT can also carry risks, such as importing viral communities, endotoxins, together with the necessary microbiota, and these could be the major barriers to translation from animal models to clinical practice (Yu et al., 2025). In the few available reports on humans, FMT safety reduced blood pressure, but the effect was not sustainable in patients with hypertension (Fan et al., 2025; Zhong et al., 2021), and reduced total and low-density lipoprotein cholesterol in patients with diabetes (Ng et al., 2022). Despite the lack of clinical studies exploring the feasibility of FMT for the treatment of myocarditis or cardiomyopathy, Hu et al. (2019) reported that FMT rebalanced the gut microbiota and attenuated myocarditis in an experimental autoimmune myocarditis (EAM) mouse model. While FMT ameliorated myocarditis in our research. These divergent results highlight the significant yet underexplored potential of FMT for myocarditis treatment, likely attributable to differences in model establishment. EAM was induced by the *α*-cardiac myosin heavy chain sequence, while VMC was caused by CVB3, an enteric virus, and our model fits better with the natural pathology after the virus infection in humans. Regarding the echocardiography data, we noticed a phenomenon. Typically, normal LVEF in mice is approximately 70–85%, whereas in the VMC model it decreases to approximately 40–60%. However, in our research, LVEF in the VMC group remained relatively high, and LVEF in the VMC-FMT group approached or even exceeded that of the control group. These observations may be attributed to several factors: 1. 4–5-week-old mice were used in our study, and due to their youth, they may possess stronger cardiac compensatory capacity. 2. The VMC model was established using a low-dose viral induction protocol; mild-to-moderate myocardial injury was induced rather than fulminant myocarditis, thus no dramatic reduction in LVEF was observed. 3. FMT exerted significant cardioprotective effects in this study; it markedly alleviated myocardial inflammatory infiltration and improved myocardial systolic function. Heart function was assessed at 14 days after modeling, by which time the FMT intervention had fully exerted its reparative effects. Nevertheless, increasing number of mice in future research may be a good solution to this issue.

According to 16S rDNA sequencing analysis, FMT increased bacterial richness and diversity, decreased the abundances of Bacteroidetes, Streptococcus, Pseudomonas, and Ralstonia, and increased the abundance of Firmicutes, bringing it close to that of the healthy control group. These data indicated that FMT could restore or reconstruct gut microbiota homeostasis, thereby improving the overall health status of the recipient in VMC mice. Specifically, elevated levels of the p_Actinobacteria and reduced levels of the p_Bacteroidota were observed in the VMC + FMT group compared with the VMC group. Consistent with our results, decreased Actinobacteria abundance and increased Bacteroidetes abundance have been reported in rats with heart failure (Zhang et al., 2025). Therefore, the improvement in cardiac function by FMT in VMC may benefit from those alterations of gut microbiota. Furthermore, Bacteroidetes can produce LPS and promote the secretion of proinflammatory mediators (e.g., IL-1, IL-6, and TNF-α) in macrophages and dendritic cells, thereby exacerbating endotoxemia (Zhang et al., 2025; Mansour et al., 2024). Thus, the reduced abundance of p_Bacteroidota may be attributable to the cardiac inflammation-alleviating effect of FMT treatment.

Furthermore, the aforementioned microbiota, such as Streptococcus, may promote VMC progression. Previous studies have shown that group A streptococcal M protein can induce inflammatory heart disease because group A streptococcal M protein, CVB3, and cardiac myosin have immunologic similarities (Huber and Cunningham, 1996). Additionally, T lymphocytes obtained from VMC mice gave an immunodominant proliferative response to the streptococcal M5 protein (Huber and Cunningham, 1996). These results suggest that the immune response to Streptococcus is important to the development of cardiac inflammation and that dampening this response may alleviate myocardial pathology. Another notable observation from the PCoA and metabolomic PCA plots (Figures 3, 5) is the high degree of dispersion among samples within the VMC group, indicating significant biological variability. Potential causes include, but are not limited to: (i) inter-individual differences in susceptibility to the modeling procedure (e.g., viral infection) and (ii) inherent biological variability of gut microbiota and metabolome, which are easily influenced by the housing environment, circadian rhythms, diet, water intake, and cage location. Increasing the number of mice would help mitigate this variability.

The gut microbiota acts as an endocrine organ, producing a wide range of bioactive metabolites (Luqman et al., 2024). Our results demonstrated profound changes in the cardiac metabolome, marked by an increase in 21 metabolites and a decrease in 12 metabolites following FMT treatment, which were mainly enriched in the steroid hormone biosynthesis pathways. These metabolites included but were not limited to desoxycortone, 3,5-dichlorosalicylic acid, 21-deoxycortisol,5-bromopyridine-3-carboxylic acid. In our previous study, hormones and hormone-related compounds such as desoxycortone were over-represented in the VMC group. This finding prompted our attention because myocarditis is more common and more severe in men than women, with a sex ratio ranging from 1:2–4 (female to male), suggesting that sex hormones may play an important role in the pathogenesis of VMC (Nguyen and Wu, 2022; Fairweather et al., 2013; Fairweather et al., 2023). According to previous reports, through estrogen receptors, 17β-estradiol signaling can inhibit reactive oxygen species-induced cardiac damage, prevent cardiac hypertrophy and apoptosis in cardiomyocytes, and delay cardiac remodeling and fibrosis (Regitz-Zagrosek, 2006). Male BALB/c mice with VMC have a stronger Th1 response, while females have a stronger Th2 response. In male myocarditis, an increase in CD11b^+^TSPO^+^ cells in the heart and an enhanced IL-18-induced Th1 response through macrophages were observed (Fairweather, 2015). These data implied that the steroid hormone biosynthesis pathway may play a vital role in alleviating myocarditis via FMT through immune regulation.

Accumulating evidence has demonstrated that gut bacteria can regulate metabolite production and that microbiota-derived products influence immune function (Xie et al., 2025). Thus, we further explored several major innate and adaptive immune cells confirmed to be involved in VMC pathogenesis (Tschöpe et al., 2020). Results showed that FMT decreased the percentages of Th1, Th17, and M1 macrophages and increased the proportions of Th2, Tregs, and M2 macrophages. Consistent with our findings, in intestinal inflammation, FMT improved chicken growth performance by balancing Th17/Treg cells (Ma et al., 2023). Similarly, in colorectal cancer mice, FMT alleviated disease progression and inflammatory responses by inhibiting Th1 and Th17 cells (Song et al., 2024). In atherosclerosis, FMT reduced aortic inflammation, inhibited M1 polarization, promoted M2 macrophage polarization, and restored the M1/M2 polarization balance (Liu et al., 2025). These data indicate that FMT had an effect on major innate and adaptive immune cells and their cytokines, thus modulating host immune homeostasis in VMC; further research is required to investigate the underlying mechanisms. Furthermore, the relatively large standard errors (SEM) for IL-10 and CD206 were found, likely attributable to inherent biological heterogeneity in the VMC animal model and the limited sample size (*n* = 5–7). Increasing the number of mice would be a viable solution to this issue.

Antibiotics might enhance immune responses by affecting gut microbiota (Lewis et al., 2015). Our results revealed significant metabolic and immune disorders in the VMC-ABX group, including elevated levels of chenodeoxycholic acid and Th1 cells. Consistent with the findings of Feng et al. (2025), antibiotic administration induced profound alterations, strengthened the pro-inflammatory signature, and promoted a Th1-skewed phenotype. Furthermore, our data revealed that antibiotics increased LVEF in the VMC-ABX group; these findings differ from those of a phase II trial, in which antibiotics failed to improve LVEF in patients with heart failure (Awoyemi et al., 2021). In line with our results, it is reported that antibiotics alleviated the condition of ASD by affecting the gut microbiota (Braakman et al., 2018), while antibiotics may have positive or negative effects in the treatment of IBD and IBS (Lee et al., 2020). Regardless of the disease context, antibiotics act as a double-edged sword, with potential benefits and risks that need to be carefully weighed.

## 5 Limitations

Our present study has some limitations. First, our experiment was based on a mouse model of VMC, and these findings need to be further supported by relevant clinical trials. Furthermore, increasing the sample size in future studies could help reduce the biological variability observed in the VMC group. Second, regarding biomarker microbiota such as Streptococcus, we observed alterations in metabolic profiles and immunity responses following FMT/ABX treatment; however, we did not explore the downstream metabolic or immune pathways and mechanisms, or provide causal evidence. Future study is needed, such as focusing on how specific changes in the microbiota directly cause alterations in specific metabolites. And how do these metabolites act on immune cells? In addition, we only observed the acute stage of myocarditis and lacked late-stage myocarditis, such as dilated cardiomyopathy (DCM). In the near future, we plan to conduct studies on chronic myocarditis and DCM to further deepen our knowledge of the mechanisms by which the gut microbiota affects VMC.

## 6 Inclusion

In our study, we investigated how fecal microbiota transplantation (FMT) and antibiotics affect the gut microbiota to control myocarditis, particularly in disturbances of cardiac metabolism and immunity. The results indicated that remodeling the gut microbiota and its metabolites via FMT could influence immune and inflammatory status and may be an effective approach to ameliorate VMC. Future studies should investigate the underlying mechanisms of immune-inflammation regulation with these bacteria-derived metabolites following FMT treatment.

## Data Availability

The raw data generated in this study can be found at: https://www.ncbi.nlm.nih.gov/, accession PRJNA1434033.

## Footnotes

1
http://ccb.jhu.edu/software/FLASH/

2
https://www.arb-silva.de/

3
http://www.drive5.com/usearch/manual/uchime_algo.html

4
http://drive5.com/uparse/

5
http://www.arb-silva.de/

6
http://www.drive5.com/muscle/

7
http://www.kegg.jp/kegg/compound/

8
http://www.kegg.jp/kegg/pathway.html

## Glossary

VMC: viral myocarditis
DCM: dilated cardiomyopathy
CVB3: coxsackievirus group B type 3
FMT: fecal microbiota transplantation
ABX: antibiotics
EAM: experimental autoimmune myocarditis
16S rDNA: 16S ribosomal DNA
UPLC-MS/MS: untargeted ultra-performance liquid chromatography–tandem mass spectrometry
PBS: phosphate-buffered saline
H&E: hematoxylin and eosin
ASVs: amplicon sequence variants
LEfSe: linear discriminant analysis effect size
LDA: linear discriminant analysis
PCA: principal component analysis
HCA: hierarchical cluster analysis
MI: myocardial infarction
LVESD: left ventricular end-systolic diameter
LVEDD: left ventricular end-diastolic diameter
LVEDV: left ventricular end-diastolic volume
LVESV: left ventricular end-systolic volume
LVEF: left ventricular ejection fraction
LVFS: left ventricular fractional shortening
