# Supplementary material for: Gut microbiota alters cardiac metabolism and immune system composition in viral myocarditis mice
Source: Front Microbiol. 2026 Jun 5;17:1828423. doi: 10.3389/fmicb.2026.1828423 (PMC13280975; doi:10.3389/fmicb.2026.1828423)
Supplement: Supplementary file 1 [file Supplementary_file_1.docx]

Supplementary Material

#
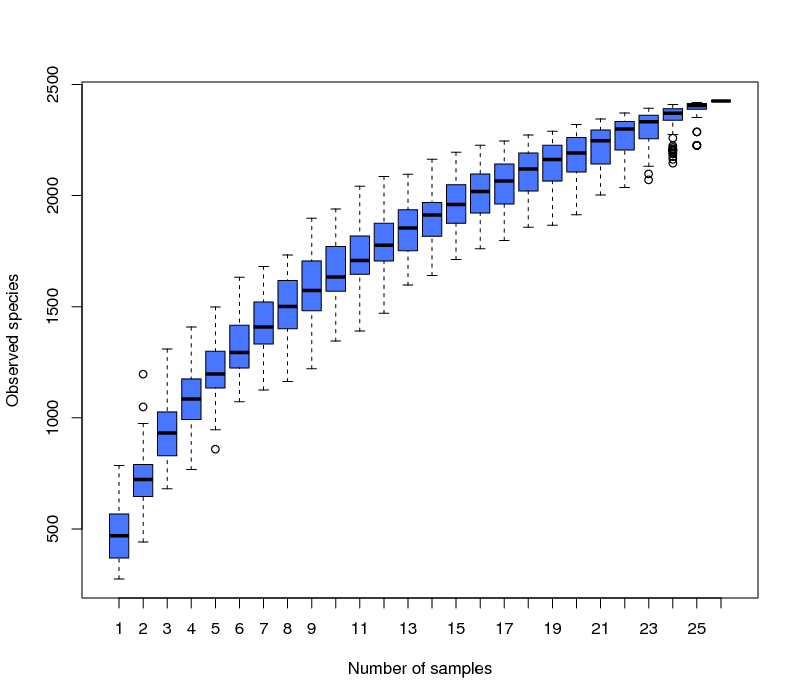


**Supplementary Material:** Species accumulation boxplot indicated the sufficiency of sample size in our study.
